# A Dynamic Model for Stem Cell Homeostasis and Patterning in *Arabidopsis* Meristems

**DOI:** 10.1371/journal.pone.0009189

**Published:** 2010-02-12

**Authors:** Tim Hohm, Eckart Zitzler, Rüdiger Simon

**Affiliations:** 1 Department of Medical Genetics, University of Lausanne, Lausanne, Switzerland; 2 Swiss Institute of Bioinformatics, University of Lausanne, Lausanne, Switzerland; 3 Computer Engineering and Networks Laboratory, Zürich, Switzerland; 4 Institute of Genetics, Heinrich-Heine University, Düsseldorf, Germany; Lund University, Sweden

## Abstract

Plants maintain stem cells in their meristems as a source for new undifferentiated cells throughout their life. Meristems are small groups of cells that provide the microenvironment that allows stem cells to prosper. Homeostasis of a stem cell domain within a growing meristem is achieved by signalling between stem cells and surrounding cells. We have here simulated the origin and maintenance of a defined stem cell domain at the tip of *Arabidopsis* shoot meristems, based on the assumption that meristems are self-organizing systems. The model comprises two coupled feedback regulated genetic systems that control stem cell behaviour. Using a minimal set of spatial parameters, the mathematical model allows to predict the generation, shape and size of the stem cell domain, and the underlying organizing centre. We use the model to explore the parameter space that allows stem cell maintenance, and to simulate the consequences of mutations, gene misexpression and cell ablations.

## Introduction

Growth of the aerial parts of higher plants relies on a life-long supply with cells by the shoot apical meristem (SAM). The SAM contains a small population of non-differentiating stem cells in the central zone at the meristem tip [1]. After cell divisions in the stem cell domain (SCD), daughter cells are shifted towards the surrounding peripheral zone, where organ primordia are initiated and cells can enter a differentiation pathway. The architectural makeup of flower primordia, which gives rise to the plant's reproductive organs, resembles that of the SAM with the main difference that stem cell activity is switched off in flowers after generation of a species-specific number of organs. It is evident that land plants such as trees, which can grow in size and produce new organs for hundreds of years, must have developed robust regulatory systems that enable them to maintain active stem cell populations also under changing or adverse environmental conditions. Disturbing stem cell regulation can arrest the growth of a plant's shoot tip, or may result in gross tissue overproliferation and failure to reproduce. More subtle alterations in stem cell proliferation can affect overall size of a seed-producing inflorescence structures, such as a maize cob, the size of a fruit, or the number of petals in a horticultural flower. We are only just beginning to understand how the fate of the stem cell population is regulated in higher plants.

Maintenance of the undifferentiating stem cell population depends on signals from cells of the organizing centre or OC, which reside underneath the SCD in a deeper region of the meristem. Several gene products have been identified that enable these adjacent cell groups to communicate with each other. The stem cells of *Arabidopsis thaliana* secrete the CLAVATA3 (CLV3) peptide, consisting of 12 amino acids [2], [3], [4]. CLV3 was shown to interact with the LRR-receptor kinase CLAVATA1 (CLV1) that is expressed in and surrounding the OC [5], [6]. A second receptor system composed of the LRR-protein CLAVATA2 (CLV2) and the membrane associated kinase CORYNE (CRN) is more widely expressed in the meristem and vasculature, and also contributes to signal perception [7], [8]. CLV3 dependent activation of the two receptor systems represses the expression of WUSCHEL (WUS), a homeodomain transcription factor that is normally produced from OC cells, and which is required for the maintenance of stem cells [9], [10]. WUS itself acts non-cellautonomously to promote stem cell fate at the meristem tip. The WUS protein does not seem to move, and it could control the expression of other genes that generate a diffusible signal which ultimately promotes stem cell identity [11]. Searches for target genes showed that several *ARABIDOPSIS RESPONSE REGULATOR* (*ARR*) genes, which are negative regulators of cytokinin signalling, are repressed by *WUS*, thus involving cytokinin in meristem maintenance [12]. However, *WUS* induces stem cell fate only at the meristem tip, and not in the (*WUS* expressing) OC cells or other surrounding cells, indicating that a spatially restricted cofactor, or a competent cellular state is required to respond to *WUS* activity [13].

Because stem cells signal back to the OC via CLV3 and its receptors to restrict *WUS* expression, a feedback circuitry is established that maintains a stable stem cell population. Support for this model of stem cell homeostasis comes from a number of experimental observations: 1) loss-of-function mutants of *WUS* cannot maintain stem cells [10]; 2) loss-of-function mutants of *CLV3* (or *CLV1*, *CLV2* or *CRN*) allow for less restricted *WUS* expression and production of excessive stem cells [3], [4], [8], [14]; 3) constitutive high level expression of *CLV3* represses *WUS*, causing stem cell loss [4]; 4) when *WUS* expression is uncoupled from repression by *CLV3*, e.g. when controlled from a heterologous promoter, the stem cell domain expands [15], [16]; 5) the *CLV3/WUS* circuitry is capable of self-organization. This was revealed by laser ablation experiments in tomato, showing that after elimination of both SCD and OC, new domains of *WUS* expression are generated at peripheral sites that then initiate new SCDs, which support further growth of the SAM [17].

However, all previous studies performed on various mutants or constitutive misexpression lines did not allow studying the immediate consequences of system perturbations. Cell ablation experiments are further complicated by wounding effects, and ectopic cell divisions in the SAM which are required for regeneration. Analyzing the dynamics of the *CLV3/WUS* circuitry at a shorter timescale required rapid and transient perturbations of gene expression. In a first study of this type, *CLV3* expression was silenced by Dexamethason-induced expression of a foldback *CLV3* RNA [18]. Live imaging of the SAM before and after *CLV3* silencing showed that expression of a *CLV3:GFP* transgene, acting as a reporter for stem cell identity, extended into cells adjacent to the central zone within 24 hours after induction. Importantly, this re-specification of peripheral cells to stem cell identity was not preceded by cell divisions. In a similarly designed experiment, induction of high level *CLV3* expression downregulated both *WUS* expression, and the stem cell marker *CLV3*, within 3 hours [14]. Together, these experiments showed that the *CLV3/WUS* circuitry is acting throughout development, and that the output, stem cell number, can be continuously readjusted in response to changing amounts of the signalling components. In line with this, fluctuations of central zone size were observed, indicating continuous activity of the circuitry [18].

However, the *CLV3/WUS* circuitry was also found to be surprisingly robust and to tolerate changes in *CLV3* expression levels over a tenfold range [14], indicating that stem cells do not directly communicate their number via the amount of released CLV3 signal. Furthermore, while strong *CLV3* signalling rapidly repressed *WUS* expression, a slowly acting compensation mechanism appeared to upregulate *WUS* with time. The components of this compensatory circuitry are unknown, but may be found among the gene set that controls *WUS* expression. *SPLAYED* (*SYD*) encodes a *SNF2*-type chromatin-remodelling ATPase that is required for *WUS* transcription [19]. BARD1, carrying BRCT and RING domains, interacts with and antagonizes SYD to restrict *WUS* expression to the OC [20]. *HANABA TARANU* (*HAN*), coding for a GATA-transcription factor, represses *WUS* postembryonically from the developing vasculature [21]. The interplay between these components is not understood, and they may act exclusively to establish a discrete *WUS* expression domain when meristems are generated. During development, a second feedback mechanism could operate via the cytokinin signalling pathway. *WUS* represses the expression of several *ARRs* in the meristem, which restrict cytokinin signalling [12]. In turn, continuous activation of *ARRs* arrests meristem activity and *WUS* expression, suggesting that *WUS* and *ARRs* mutually repress each other.

We have generated a computational model of stem cell fate regulation by the *CLV3/WUS* circuitry in the shoot apical meristem. Our model incorporates two feedback regulatory systems that merge upon *WUS* regulation. The driving force for modelling was to better understand the forces that shape the *CLV3* and *WUS* expression domains, while making the minimal number of necessary assumptions about the factors to be involved. We used the model to study the effects of targeted system perturbations, and to explore the parameter space that allows for stem cell homeostasis under fluctuating conditions.

## Results

### Model Components and Basic Assumptions

**Figure 1:**
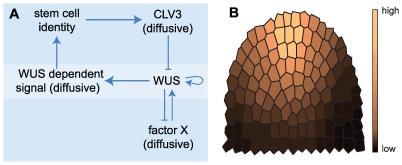
Modelling components and cellular framework. (A) Components and their interactions regulating stem cell homeostasis in *Arabidopsis*.(B) Two-dimensional meristem frame with assumed anchoring distribution indicated by colouring.

We propose a partial differential equation (PDE) model to follow the dynamics of gene regulation across the SAM (Fig. 1A). Conceptually, at the centre of the model lies the regulation of *WUS* via two separable feedback operated reaction-diffusion systems, a commonly used type of differential equation models for developmental processes in biology [22]. Instead of representing the entire meristem structure, we here restrict the spatial component to two dimensions using an artificial longitudinal section through the SAM (Fig. 1B). Cells within this meristem section are modelled as discrete entities. We neglected growth and cell divisions for two reasons: firstly, we are concentrating on meristem homeostasis, i.e. meristem size remains unaltered, and secondly, the gene regulation that is considered in this study is faster than the cell cycle. The cellular or tissue framework thus remains static. The regulative processes within cells are mapped to a set of PDEs constituting a gene regulative program, which is executed in each cell. The components and underlying assumptions of our model are summarized as follows (Fig. 1A):

#### Stemness

Cells of the meristem can acquire stem cell identity, reflected in their level of *stemness*, which is controlled by a *WUS*-dependent signal (*WUS-signal*, see below). We avoided an artificial and static cut-off concentration for *WUS-signal*, above which cells switch to the stem cell status, and instead established a dynamic but sigmoidal response to *WUS-signal*, that results in variable levels of *stemness* to represent a cell's state.

Experimental evidence from WUS misexpression shows that only outer cell layers acquire stem cell identity. The underlying factors responsible for this are not known. We are now not postulating another signal, but take this observation into account by allowing only cells in outer cell layers to acquire stem cell identity. Therefore, only cells in the outer layers of the meristem are competent to react to *WUS*-signal and we restricted *stemness* to the outer layers. Stem cells express the signalling molecule CLV3, proportional to their *stemness* level. The *stemness* levels are expressed by the model variable [*st*].

#### CLV3

CLV3 freely diffuses to neighbouring cells. To avoid flooding the entire model with CLV3, the CLV3 peptide is regarded to decay with time. We eliminated the need for receptor proteins or other signalling components because insufficient quantitative data are available to assess their contribution. Furthermore, CLV-signalling appears to be largely controlled by the amount of available CLV3 peptide [14]. Thus, the local CLV3 concentration is computed to directly restrict WUS expression. The *CLV3* levels are expressed by the model variable [*CLV3*].

#### WUS

**Figure 2:**
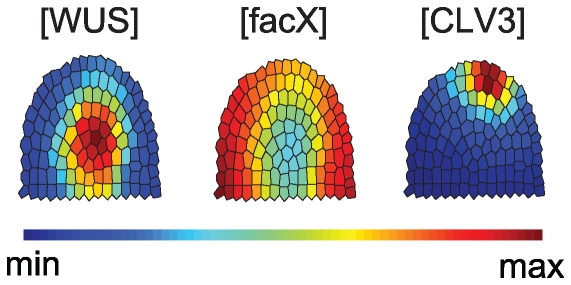
System without anchoring distribution. Equilibrium state of a simulation where the anchoring distribution guiding *WUS* expression is exchanged by a constant *WUS* reaction rate. As a result of the missing positional information, SCD and OC keep their positions relative to each other, but the SCD is now formed at a random location in the outer meristem layers.

We assume that WUS protein is not mobile and therefore remains mostly in the cells where the *WUS* gene is expressed [11], except for weak leakage diffusion. While all cells of the model meristem are in principal able to express *WUS*, we added a spatial parameter which makes cells that reside closer to the meristem tip more competent to activate *WUS* expression (Fig. 1B). This anchoring was found to be necessary in our model to ensure correct positioning of the two functional domains (SCD and OC) within the dome. Without the spatial component, immediately neighbouring SCD and OC are still formed, but at more random locations (Fig. 2). The requirement for a spatial component reflects the fact that our virtual meristem is not structured, i.e., all cells are intrinsically equal and carry no positional information. In plant meristems, such spatial information will be provided by signals within and between cell layers, or from the vasculature. We introduced a positive feedback loop for *WUS* via autoactivation. Although not experimentally proven, it is supported by the observation of a rapid upregulation of *WUS* expression in regenerating callus [23]. *WUS* promotes the expression of *WUS-signal*, which is mobile and can diffuse to neighbouring cells. The *WUS* levels are expressed by the model variable [*WUS*].

#### WUS-signal

*WUS-signal* is generated by all *WUS* expressing cells, and the amount produced depends on the levels of *WUS* expression. Similar to *CLV3, WUS-signal* is mobile and degraded at a constant rate. Cells react to the amount of *WUS-signal* they receive with *stemness*. Only outer cell layers of the meristem are competent to respond to the *WUS-signal*. The *WUS-signal* levels are expressed by the model variable 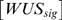.

#### Factor X

To account for *CLV* independent regulation of *WUS* expression, we incorporated a *factor X (facX)*. At the start of the simulation, *facX* is expressed homogeneously in all cells and is freely diffusing. *FacX* induces *WUS* expression, but is itself under negative feedback regulation by *WUS* [14]. This is implemented through active degradation or consumption of *facX* by *WUS*. The interactions between *WUS* and *facX* are thus based on an activator-substrate-like mechanism that will generate a discrete WUS domain. The *facX* levels are expressed by the model variable [*facX*].

The described entities are compiled into a PDE representation of the intracellular gene regulative program given by Eqs. (0.1)–(0.5) (see Materials and Methods section). An overview of the interactions is given in Fig. 1A.

**Figure 3:**
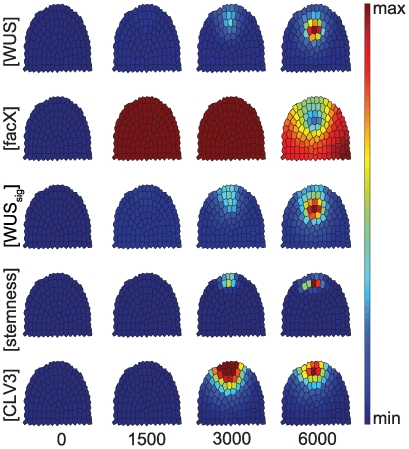
Time course simulation of a wild-type meristem. Grading from blue (minimum) to red (maximum) illustrates the relative concentration of the indicated components. From left to right, the simulations were developed from close to zero concentrations for number of steps shown below, until the (stable) equilibrium state was reached.

The resulting model depends on a set of parameters like kinetic constants; validation of our model therefore required first to identify a parameter setting that allow reproducing the two functional meristem domains, i.e. the *CLV3*-expressing SCD and the *WUS*-expressing OC, at approximately those locations which are experimentally observed in wildtype meristems (Fig. 3). The model parameters have been tuned by hand using a decomposition of the model (see Materials and Methods section).

### Simulation of Wildtype

Starting from almost zero concentrations of all considered components, the system was simulated until an equilibrium state was reached (Fig. 3); since we investigate system behaviour by means of numerical integration of the model equations, under ‘equilibrium state’ we understand a state where all derivates are zero or reasonably close to zero. In the wild-type scenario, a given meristem showed *WUS* expression first at the meristem tip, triggered by a sufficiently high level of *facX*. *WUS* then increased, thereby repressing *facX* at the same location. The centre of the OC shifted downwards. Distribution of *WUS-signal* overlaps with that of *WUS*, but since *WUS-signal* is diffusible, it is located in a wider domain and extended always to the meristem tip. Together with an increase of *stemness* at the meristem tip, *CLV3* became expressed, pushing the OC downwards from its initial position. This time course reproduced the dynamic changes in gene expression patterns that are observed during embryonic development of the shoot meristem.

### Simulations of mutants and system perturbations

#### I. Reducing CLV3 expression

We next tested the consequences of reducing *CLV3* expression. When *CLV3* is downregulated during plant development, both the SCD and the OC expand laterally due to unrestricted *WUS* expression [4], [16], [18]. Furthermore, *WUS* expression is then no longer excluded from the meristem tip.

**Figure 4:**
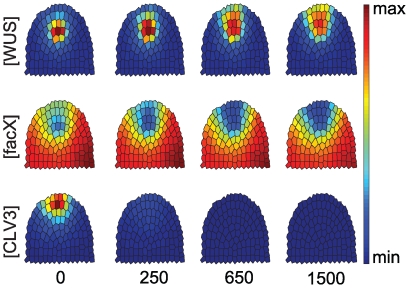
Time course simulation for a clv3 loss-of-function mutant. Only the concentrations of *WUS*, *facX* and *CLV3* are shown, starting from an equilibrated wild-type (0). At calculation time point 1, *CLV3* concentration is set to 0, and simulations proceed until equilibrium. Note that the *WUS* domain expands and shifts upwards.

Our in silico analysis started from an equilibrated wild-type meristem, thus simulating a conditional knock-out of *CLV3*. When *CLV3* expression was stopped, the SCD rapidly enlarged due to recruitment of lateral cells. At the same time, the OC expanded and shifted towards the meristem tip (Fig. 4). A similar, but less pronounced effect was seen when *CLV3* was still expressed, but at reduced levels (see Fig. S1 and Text S1).

In addition, we simulated the effects of reducing CLV3 receptor activity, i.e. CLV1 or CLV2 and CRN constructs (see Fig. S2 and Text S2). These simulations showed behaviour comparable to reductions in *CLV3* expression and thereby supports the assumption that from a modelling perspective it is sufficient to model *CLV3* activity as a representative for *CLV* signalling.

#### II. Increasing CLV3 expression

**Figure 5:**
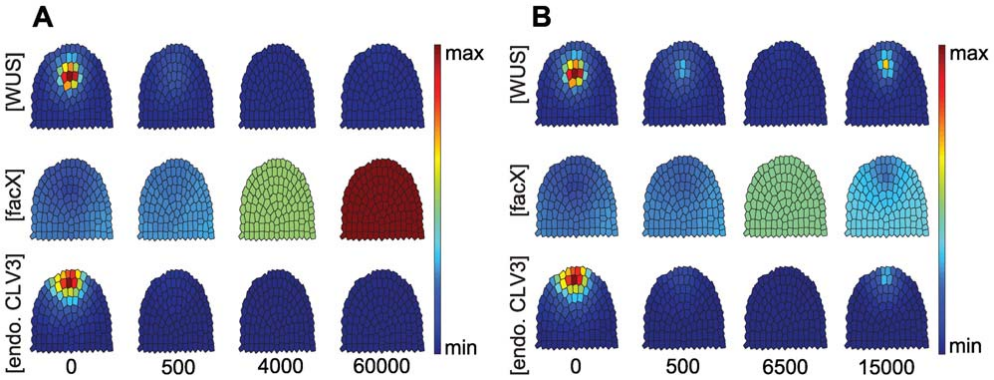
Response to CLV3 overexpression in the entire meristem. Time-evolution of *WUS*, *FacX* and *CLV3* concentrations upon strong (A) or intermediate (B) level overexpression of *CLV3* is shown, starting from equilibrated wild-type meristem (time-point 0) until the simulations reach a new equilibrium state. *endoCLV3* = *CLV3* expression from the endogenous promoter, taken as reporter for stem cell identity. Note that both a *WUS* expression domain and stem cells are reinitiated in (B), but not in (A).

Plants that continuously express *CLV3* fail to maintain a shoot meristem due to an early arrest of *WUS* expression and stem cell differentiation [4]. However, inducible overexpression during development was found to be compensated in some flower meristems, resulting in a recovery of *WUS* expression at later stages [14]. In our simulations, high level expression of *CLV3* in all cells caused a rapid shrinkage of the OC, downregulation of *WUS*, and a reduction in *stemness*, concomitant with a reduction in *CLV3* expression levels from the SCD (Fig. 5A). Overexpression of *CLV3* set at an intermediate level resulted in *WUS* repression and a rapid loss of *stemness*, which recovered with time (see Fig. 5B). A similar behaviour was observed in plant floral meristems in response to induced overexpression of *CLV3* [14]. Low level overexpression of *CLV3* allowed the system to reach a new stable equilibrium state, with a smaller OC and SCD (see Fig. S3 and Text S3).

We further tested the robustness of the system against perturbations by analyzing the response to altered endogenous *CLV3* expression in small, discrete steps: the effectiveness of *stemness*-dependent *CLV3* expression is tested in a range from 10% to 620% in 10% steps. Varying *CLV3* levels from 90% to 620% compared to wildtype affected the size of the SCD, while OC cell number remained constant (see Fig. S1B). This indicates that OC and SCD sizes are not strictly coupled, which has been also noted experimentally when analyzing the sizes of OC and SCD in plants grown under diverse environmental conditions [24]. In the simulations, we varied *CLV3* expression in 10% steps in a range from 10% to 620%.

#### III. Altering WUS expression

**Figure 6:**
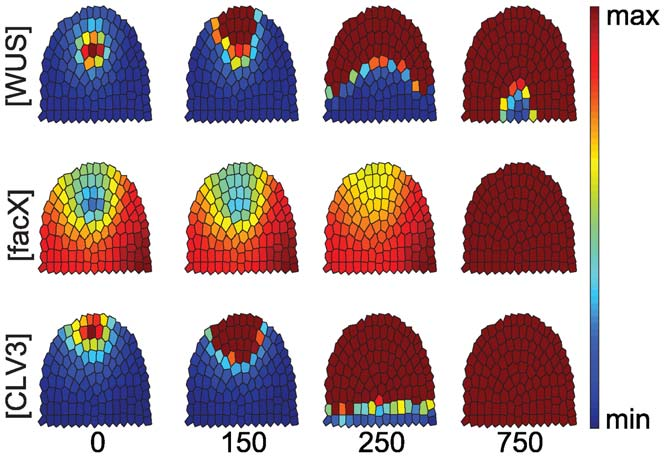
Simulation for WUS misexpression in the stem cell domain. Starting from an equilibrated wild-type meristem, misexpression of *WUS* from the *CLV3* promoter (*CLV3*≫*WUS*) is simulated until a new equilibrium state is reached. Cells in the meristem now aquire mixed identities and express both the OC and SCD marker genes.

Lowering *WUS* expression levels reduces the sizes of both SCD and OC to a similar extent, and will cause a loss of both domains when *WUS* is fully repressed (see Fig. S4 and Text S4). Ectopic *WUS* expression was tested by changing the effect of *CLV3* signalling on *WUS* activity from repressive to activating. In plants, this has been achieved by expressing *WUS* from the *CLV3* promoter [15], which caused the coalescence of OC and SCD at the meristem tip together with lateral expansion of this joint domain. This cell behaviour is also observed in our simulations (Fig. 6).

#### IV. Regeneration and de-novo generation of OC and SCD

**Figure 7:**
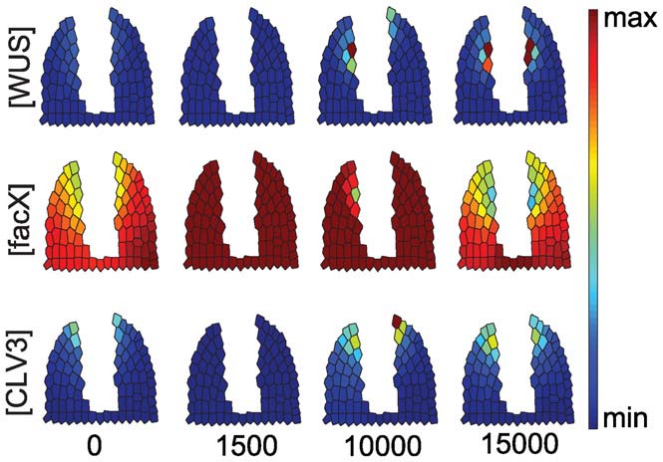
Simulation for a cell ablation scenario. The central region of the meristem was eliminated by virtual cell ablation. Note that even in the absence of an SCD and underlying OC, both domains can be partially restored.

After ablating SCD and OC from the meristem by pointing a laser beam at the meristem tip, *WUS* becomes expressed at the periphery, and the OC and SCD are regenerated with time [17], highlighting that cells at the periphery are capable of, but normally inhibited from the acquisition of OC identity. We simulated the laser ablation experiment starting from an equilibrated wildtype meristem, and eliminated all *WUS* or *CLV3* expressing cells from the meristem. We found that a new OC was generated which induced a SCD nearby (Fig. 7). This shows the self-generative capacity of our meristem model. During normal plant development, new meristems are generated during embryogenesis, flowering, and when axillary meristems are initiated. By simply altering *facX* expression levels in a given cellular framework, we could simulate the generation of a new OC and SCD, which coordinated increase in size when *facX* is further upregulated (see Fig. S5 and Text S5).

#### V. Role of facX and WUS feedback regulation

**Figure 8:**
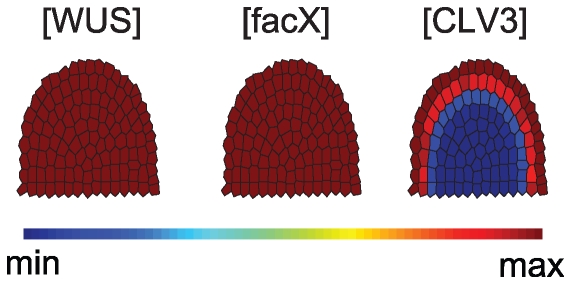
System neglecting feedback of WUS on facX. Archetype system behaviour for simulations without feedback of *WUS* on *facX*: the meristem overproliferates, *WUS* is expressed in all meristem cells and all cells in the outer layers acquire stem cell identity indicated by their expression of the stem cell marker *CLV3*.

In addition to the already described scenarios that are all inspired by previously conducted experiments, we analysed the role of *facX* and the interaction of *WUS* with *facX*. Without feedback of *WUS* to *facX*, *facX* could be exchanged for a constant expression of WUS. We tested this idea by eliminating the feedback term of *WUS* on *facX* (see Eq. (0.2) in Materials and Methods). However, using an evolutionary algorithm to search the parameter space of the resulting model, we could not identify any parameter settings resulting in the desired system behaviour, with separate but adjacent SCD and OC. We therefore conclude that *facX* and the feedback between *WUS* and *facX* are vital for the model to initiate stable Turing patterning. Candidates to realize the role of *facX* are genes and functions that control WUS expression. Because eliminating *facX* destabilizes the OC, we predict that mutations in genes contributing to *facX* function should result in either unrestricted WUS expression and meristem overproliferation (Fig. 8), or meristem arrest.

#### VI. Influence of anchoring factor distribution on system behaviour

We have introduced artificial positional information in form of an anchoring distribution (Fig. 1B) to stimulate SCD and OC positioning at the meristem tip. To test the possible influence of this anchoring distribution on the pattern formation capabilities of the model, we exchanged the distribution for a constant value. Although positioning of SCD and OC became more variable now, we were still able to identify parameter sets that result in spatially confined and adjacent SCD and OC (Fig. 2), indicating that the anchoring distribution has no significant influence on the qualitative behaviour of our model.

## Discussion

Mathematical modelling is a tool that allows asking the most stringent questions concerning the dynamic behaviour of predicted gene regulatory networks; it also quickly uncovers the restrictions and shortcomings of assumed interaction maps, and thus provides guidance to direct future experiments. We had initially attempted to build a model for the SCD and OC, based solely on the interaction between two activator-inhibitor based systems (*CLV3/WUS*, and *WUS/facX*) which were linked via *WUS* as the common node. Conceptually, the underlying assumption was that SCD and OC could originate independently of each other, but that their maintenance and relative position are controlled by mutual feedback regulation. However, such a model failed to reproduce the domain arrangement observed in actual meristems for the model's parameter space which we explored using a stochastic parameter estimation technique, namely an evolutionary algorithm [25]. For the evolutionary algorithm, we used an objective function described previously [26], extended by a simple domain recognition procedure capable of identifying circular domains. This indicates that an essential component was missing from this model. The most common outcome which we achieved was not juxtaposition, but an overlap of the SCD with the OC. To improve the spatial separation of the two domains, we considered that cells within an actual meristem differ from each other by their position. Several misexpression experiments using *WUS* had previously shown that the cellular response to *WUS*-derived signals depends on a cells' relative position within the meristem, corresponding to its developmental trajectory. Only by adding spatial components to our model we were able to achieve a realistic sizing and arrangement of the two domains within the meristem; removal of this spatial component causes extensive spatial overlapping of the SCD and OC.

Jönsson et al. had previously described a model for the *WUS/CLV3* interaction that concentrated on the generation of the *CLV3* domain (the SCD in our model) by a *WUS* derived signal [27]. This model did not yet include the negative feedback regulation of *CLV3* signalling upon *WUS* expression, and the creation and maintenance of the *WUS* domain was not simulated. To confine the *CLV3* domain to the meristem tip, the authors proposed that an (unidentified) factor diffusing from the outermost meristem layer, the L1, together with the *WUS*-dependent signal, induced *CLV3* expression. They later used a reaction-diffusion model combined with two repressive signals, derived from the L1 and stem tissue, to activate WUS expression in a deeper meristem region [28]. Both models were successful at reproducing either the OC or the SCD, but did not incorporate the mutual interdependence between the factors that shape the two domains, and were less parsimonious with system components than the model we describe here.

A recently published model by Geier et al. [24] did not describe the spatial arrangement of domains, but addressed the observation that SCD and OC sizes vary strongly under changing environmental conditions. The model describes the SCD and OC as cell pools that are connected via differentiation rates and expand due to cell proliferation, which is regarded as an externally controlled parameter. Variation in the relative sizes of SCD and OC can be explained by assuming that a differentiation signal *X* is produced by OC or SCD, which can buffer the response of the cell pools against changes in proliferation rates. Although this model did not allow reproducing all mutant and overexpression experiments that we have simulated here, it combined modelling approaches with quantitative data, and highlighted the enormous developmental plasticity of the meristem.

We challenged our model by altering central system parameters to simulate mutant phenotypes and published transgenic experiments. In all experiments, our model proved to be robust against small-scale perturbations (see Text S6). This stability probably results from the combination of two feedback operated system, whereby one of them, the *WUS/facX* system, acts as a buffer that dampens fluctuations in *WUS* levels. Furthermore, the reaction rates that influence *WUS* expression in our simulations are one order of magnitude smaller than those controlling *CLV3* levels. Increased stability against signalling noise was uncovered in the analysis of coupled positive feedback systems if the two linked regulatory loops operated at different speeds [29], [30]. Our modelling approach has revealed that combined feedback systems are sufficient to allow the generation and robust maintenance of two distinct cellular domains in a meristem, requiring only minimal assumptions about spatial restrictions of the system. The challenges ahead are now to extend the cell model into the third dimension, and incorporate cell divisions, but also to add other regulatory networks that control organ initiation and cell differentiation, approaching the goal of a virtual meristem.

## Materials and Methods

Based on the gene interaction diagram shown in Fig. 1A, a system of coupled PDEs is set up that describes the temporal evolution of concentrations of the factors 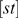, 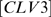, 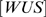, 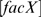 and 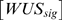 inside the cells of the SAM. For this model, the three-dimensional dome of cells constituting the SAM is restricted to a two-dimensional artificial longitudinal section (Fig. 1B). This section was generated by positioning cell centres and using a Voronoi decomposition in order to generate possible cell walls and thereby defining the cells. For the simulations we assume zero flux boundaries confining the simulated domain. The model equations are given in the following:
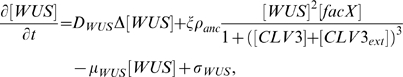
(0.1)

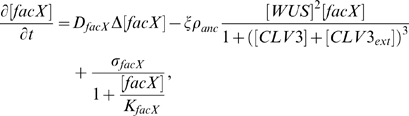
(0.2)

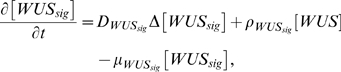
(0.3)

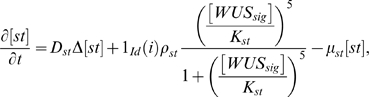
(0.4)

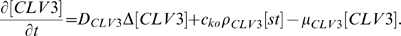
(0.5)


The model depends on the following parameters: reaction rates 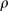, basal expression rates 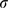, degradation rates 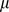, and kinetic constants 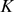. While most parameters settings are similar for all cells of the considered simulated domain, there are two exceptions: (i) the reaction rate 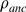 (Eqs. (0.1) and (0.2)) is given by a distribution with its maximum in the meristem tip (Fig. 1B). It is an artificial spatial component necessary for correct location of developing SCD and OC within the meristem. In addition 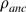 is perturbed by a small uniformly random value 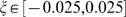 which is a random influence necessary for this subsystem to produce patterns. (ii) The reaction term guiding 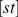 depends on the indicator function 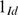. Since we assumed that only cells in the outer cell layers are competent to acquire stem cell identity, for competent cells 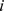 the indicator function returns a value 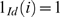 and 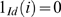 otherwise. While the former parameters refer to processes taking place within the cells, the model includes interactions between neighbouring cells as well. This interaction is modelled by diffusion terms 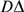, where 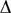 is the Laplace operator in two dimensions and 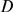 is a diffusion rate. Note that although 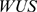 and 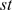 are considered to be only locally active, their model equations (Eqs. (0.1) and (0.5)) contain diffusion terms since for these two factors we assume a weak leakage diffusion.

On top of the parameters we already described, the model contains a set of parameters that is used to accommodate the different modelled mutants and experiments. In this context, 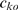 is used to simulate regulation of endogenous CLV3; 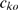 modifies the reaction term guiding 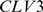 expression where 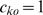 simulates a wildtype situation while 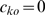 represents a knockout and values 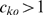 represent overexpression of endogenous 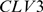. Choosing 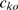 values 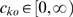, graded scenarios can be simulated. The parameter 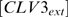 is used to simulate a *CLAVATA* background in all cells. In a wildtype scenario 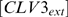 is set to 0 and with 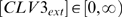 a range of graded 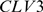 background strengths can be simulated. In addition, for simulations testing conditions with respect to 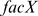 under which OC and SCD are generated the already introduced parameter 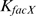 is varied.

### Parameter Calibration

The model parameter setting for the wildtype simulations as well as for the simulations of mutants are given in Table 1 and Table 2. Although the model parameters are dimensional, at this point we disregard their dimensionality: since only qualitative data is available to calibrate the model, the given parameter setting represents an equivalence class of parameter settings that can be generated starting from the given parameter setting by rescaling within the biologically feasible range. Therefore it is not possible to give exact dimensional values for corresponding biological parameters without at least some anchoring qualitative measurements.

**Table 1:** Summary of constant model parameters.

Parameter	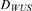	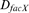	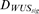	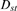	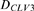	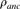	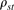	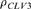	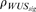
Value	0.002	0.02	0.02	0.002	0.02	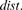	0.5	0.6	0.03
Parameter	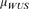	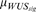	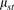	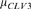	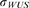	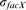	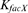	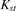	
Value	0.004	0.05	0.05	0.01	0.0002	0.004	0.2	1	

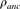
 follows a constant distribution shown in Fig. 1B.

**Table 2:** Scenario dependent model parameters with their respective values.

	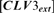	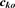
WT	0	1
Laser ablation	0	1
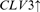 , medium	1	1
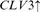 , strong	1.5	1
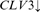	0	0
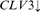 , gradual	0	0.2
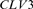 ≫ 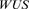	0	1

WT describes the wild type setting. 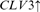: over-expression of *CLV3* in all cells (stem cells and non-stem cells). 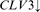: *clv3* loss of function mutant. *CLV3* ≫* WUS*: Expression of *WUS* in the stem cell domain, controlled by the *CLV3* promoter.

The model parameters have been tuned by hand using a type of hierarchical decomposition of the system: from a developmental perspective firstly the OC is formed which then induces the formation of a SCD. For the parameter tuning process we therefore divided the system into two parts. (i) Equations (0.1) and (0.2) which are responsible for the formation of the OC, and (ii) Eqs. (0.3) to (0.5) that constitute the SCD generating part of the system. During a three-stage process we started the tuning process of the model parameters by considering only the OC generating system, in order to identify parameters resulting in a single and spatially confined OC domain. For this subsystem, we started with parameter settings as documented previously (Koch and Meinhardt, 1994), and we were able to identify fitting parameters reasonably close to the initial settings. Using the resulting OC as input, we then tuned the parameters for the SCD generating part of the system. Here we ignored the feedback of 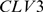 on 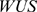 and aimed on the identification of parameters which resulted in an SCD of appropriate size and an area under 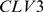 influence that extends the SCD but remains spatially confined as well. In the last step we considered the full system adjusting the parameters responsible for the feedback process between the two subsystems.

### Simulation

For the numerical simulation of the PDEs, we assume zero flux conditions on the boundaries of the cell plane. In order to simulate the time evolution of the model, the model equations are numerically integrated. The equations have to be discretized with respect to time and space, here using a constant time step 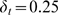 and applying a finite difference scheme in cellular resolution to the space-dependent diffusion terms. Each cell is thereby represented by its centre and for the sake of simplicity, cell volumes are considered to show the gradients that would be assumed between the concentrations simulated for the cell centres. In addition, we assume free diffusion as the only means of communication between cells. Because the precise communication underlying *WUS* dependent signalling is still unknown, free diffusion represents a sort of ‘maximum entropy choice’. In terms of modelling complexity we benefit from this fact: with free diffusion, extracellular spaces, cell walls and membranes can be neglected during the simulation process. In addition, due to the Voronoi decomposition used to generate the considered section through the meristems, cell volumes and surfaces tend to even out. Since the model is not supposed to generate quantitative data, but rather to investigate qualitative behaviour, we neglect the influence of cell surfaces and volumes during simulations.

The diffusion terms in the considered system tend to be stiff and we therefore chose to use a variant of the second order implicit Crank-Nicolson integrator as presented previously by others [31] for these terms. To the only-time-dependent terms a faster explicit Adams-Bashford scheme [31] is applied instead. This implicit-explicit method is chosen in order to reach an appropriate trade-off between necessary computational effort and simulation accuracy.

The numerical simulations for the considered scenarios are done in a two-stage process. The first stage is used to equilibrate the system starting from the initial conditions. Here, under ‘equilibrium state’ we understand a system state in which all derivates are reasonably close to zero. During the second stage the parameters are adapted in order to accommodate the considered scenarios. As initial condition for the first stage, the 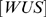 level of all cells is homogeneously initialized with a starting concentration of 0.01. All other species are initialized with a value of 0. In the first stage, the system is simulated for 30000 time units. For the second stage we use the equilibrium concentrations obtained in the first stage as initial conditions, the parameters are adapted and in case of the laser ablation scenario the tissue topology is adapted. Afterwards the system is simulated for further 15000 time steps.

### Modelling Background

To model biological systems there exists a range of different mathematical modelling approaches, e.g. stochastic molecular simulations, differential equation models, or discrete dynamic models. The available models vary with respect to possible level of detail where a gain in detail comes at the cost of additional computational effort. Since the focus of this study is to provide a model capable of reproducing the intricate dynamics underlying the maintenance of the SAM, we chose a differential equation model––an approach that provides the necessary level of detail and is commonly used to model the considered type of systems.

From a qualitative perspective, SAM maintenance is a question of developing and maintaining a patterning of a tissue with respect to distinct domains with specific gene expression profiles. A popular approach in developmental biology to capture pattern formation are reaction diffusion systems developed by Turing in the 1950s [22], [32]. Relying on diffusion, these systems are capable of producing spatially heterogeneous patterns of somewhat antagonistic reactions initiated by an initial small perturbation. Here we employ a similar mechanism: Eqs. (0.1)–(0.2) are a variant of the activator-substrate model [33] - a system that is known to produce circular domains that remain mobile and thereby allow an OC that is forming in the meristem tip to move down after initiation of the SCD. Eqs. (0.3)-(0.5) are derived guided by the law of mass action. Still, parameters like the Hill coefficients in Eq. (0.4) have been further tuned, e.g. large Hill coefficients are used in Eq. (0.4) in order to produce a sharper transition between cells showing a high level of *stemness* and neighbouring cells with low levels of *stemness*. In turn, such parameter choices reflect possible underlying biological reactions like the formation of homodimers or other forms of cooperativity.

## Supporting Information

Figure S1 Effects of reduced CLV3 levels on OC and SCD. (A) Time course simulation for a conditionally reduced CLV3 expression. (B) Impact of CLV3 expression levels on the sizes of OC and SCD. To assess the number of cells in the respective domains, the concentrations were discretized using thresholds relative to the wild type concentrations: For stemness: δ_st_ = 0.21, for WUS: δ_WUS_ = 0.31.
(1.26 MB EPS)

Figure S2 Simulating loss-of-function mutants in CLV1. (A) clv1 loss-of-function scenario (c1_ko_ = 0), (B) a simulation where CLV1 retains some activity (c1_ko_ = 0.2).
(1.69 MB EPS)

Figure S3 Gradual increase of exogenous CLV3 expression levels. (A) Time course simulation for a low level CLV3 overexpression ([CLV3_ext_] = 0.7). (B) Graded system response to different exogenous CLV3 expression levels. The [CLV3_ext_] level is varied in [0, 2]. To assess the number of cells in the respective domains, the concentrations of stemness and WUS were discretized using thresholds relative to the wild type concentrations. For stemness: δ_st_ = 0.21; for WUS: δ_WUS_ = 0.31 is used.
(1.22 MB EPS)

Figure S4 Altering WUS expression. (A) A simulation for slightly reduced WUS expression level (w_ko_ = 0.6), (B) a simulation for with intermediate WUS expression (w_ko_ = 0.4), (C) a simulation for a WUS loss-of-function scenario (w_ko_ = 0).
(2.38 MB EPS)

Figure S5 Regeneration and de-novo generation of OC and SCD. To assess the number of cells in the respective domains, concentrations were discretized using thresholds relative to the wild type concentrations. For stemness: δ_st_ = 0.21, for WUS: δ_WUS_ = 0.31 is used.
(0.46 MB EPS)

Text S1 Reducing CLV3 expression.
(0.03 MB RTF)

Text S2 Simulating loss-of-function mutants in CLV1.
(0.86 MB RTF)

Text S3 Gradual increase of exogenous CLV3 expression levels.
(0.03 MB RTF)

Text S4 Altering WUS expression.
(0.18 MB RTF)

Text S5 Regeneration and de-novo generation of OC and SCD.
(0.08 MB RTF)

Text S6 Sensitivity analysis for model parameters.
(0.17 MB PDF)

## References

[1] Stahl Y, Simon R (2005). Plant stem cell niches.. Int J Dev Biol.

[2] Kondo T, Sawa S, Kinoshita A, Mizuno S, Kakimoto T (2006). A plant peptide encoded by CLV3 identified by in situ MALDI-TOF MS analysis.. Science.

[3] Fletcher JC, Brand U, Running MP, Simon R, Meyerowitz EM (1999). Signaling of cell fate decisions by CLAVATA3 in Arabidopsis shoot meristems.. Science.

[4] Brand U, Fletcher JC, Hobe M, Meyerowitz EM, Simon R (2000). Dependence of stem cell fate in Arabidopsis on a feedback loop regulated by CLV3 activity.. Science.

[5] Clark SE, Williams RW, Meyerowitz EM (1997). The CLAVATA1 gene encodes a putative receptor kinase that controls shoot and floral meristem size in Arabidopsis.. Cell.

[6] Ogawa M, Shinohara H, Sakagami Y, Matsubayashi Y (2008). Arabidopsis CLV3 peptide directly binds CLV1 ectodomain.. Science.

[7] Jeong S, Trotochaud AE, Clark SE (1999). The Arabidopsis CLAVATA2 gene encodes a receptor-like protein required for the stability of the CLAVATA1 receptor-like kinase.. Plant Cell.

[8] Müller R, Bleckmann A, Simon R (2008). The Receptor Kinase CORYNE of Arabidopsis transmits the Stem Cell-Limiting Signal CLAVATA3 Independently of CLAVATA1.. Plant Cell.

[9] Mayer KF, Schoof H, Haecker A, Lenhard M, Jurgens G (1998). Role of WUSCHEL in regulating stem cell fate in the Arabidopsis shoot meristem.. Cell.

[10] Laux T, Mayer KF, Berger J, Jurgens G (1996). The WUSCHEL gene is required for shoot and floral meristem integrity in Arabidopsis.. Development.

[11] Gross-Hardt R, Lenhard M, Laux T (2002). WUSCHEL signaling functions in interregional communication during Arabidopsis ovule development.. Genes Dev.

[12] Leibfried A, To JP, Busch W, Stehling S, Kehle A (2005). WUSCHEL controls meristem function by direct regulation of cytokinin-inducible response regulators.. Nature.

[13] Tucker MR, Hinze A, Tucker EJ, Takada S, Jurgens G (2008). Vascular signalling mediated by ZWILLE potentiates WUSCHEL function during shoot meristem stem cell development in the Arabidopsis embryo.. Development.

[14] Müller R, Borghi L, Kwiatkowska D, Laufs P, Simon R (2006). Dynamic and compensatory responses of Arabidopsis shoot and floral meristems to CLV3 signaling.. Plant Cell.

[15] Brand U, Grunewald M, Hobe M, Simon R (2002). Regulation of CLV3 expression by two homeobox genes in Arabidopsis.. Plant Physiol.

[16] Schoof H, Lenhard M, Haecker A, Mayer KF, Jurgens G (2000). The stem cell population of Arabidopsis shoot meristems in maintained by a regulatory loop between the CLAVATA and WUSCHEL genes.. Cell.

[17] Reinhardt D, Frenz M, Mandel T, Kuhlemeier C (2003). Microsurgical and laser ablation analysis of interactions between the zones and layers of the tomato shoot apical meristem.. Development.

[18] Reddy GV, Meyerowitz EM (2005). Stem-cell homeostasis and growth dynamics can be uncoupled in the Arabidopsis shoot apex.. Science.

[19] Kwon CS, Chen C, Wagner D (2005). WUSCHEL is a primary target for transcriptional regulation by SPLAYED in dynamic control of stem cell fate in Arabidopsis.. Genes Dev.

[20] Han P, Li Q, Zhu YX (2008). Mutation of Arabidopsis BARD1 Causes Meristem Defects by Failing to Confine WUSCHEL Expression to the Organizing Center.. Plant Cell.

[21] Zhao Y, Medrano L, Ohashi K, Fletcher JC, Yu H (2004). HANABA TARANU is a GATA transcription factor that regulates shoot apical meristem and flower development in Arabidopsis.. Plant Cell.

[22] Tomlin CJ, Axelrod JD (2007). Biology by numbers: mathematical modelling in developmental biology.. Nat Rev Genet.

[23] Gordon SP, Heisler MG, Reddy GV, Ohno C, Das P (2007). Pattern formation during de novo assembly of the Arabidopsis shoot meristem.. Development.

[24] Geier F, Lohmann JU, Gerstung M, Maier AT, Timmer J (2008). A quantitative and dynamic model for plant stem cell regulation.. PLoS ONE.

[25] Foster JA (2001). Evolutionary Computation.. Nat Rev Genet.

[26] Hohm T, Zitzler E, Marchiori E, Moore JH, J.C. R (2007). Modeling the Shoot Apical Meristem in A. thaliana: Parameter Estimation for Spatial Pattern Formation..

[27] Jönsson H, Shapiro BE, Meyerowitz EM, Mjolsness E (2003). Signalling in multicellular models of plant development: Academic Press, London.

[28] Jönsson H, Heisler M, Reddy GV, Agrawal V, Gor V (2005). Modeling the organization of the WUSCHEL expression domain in the shoot apical meristem.. Bioinformatics.

[29] Brandman O, Ferrell JE, Li R, Meyer T (2005). Interlinked fast and slow positive feedback loops drive reliable cell decisions.. Science.

[30] Brandman O, Meyer T (2008). Feedback loops shape cellular signals in space and time.. Science.

[31] Ruuth SJ (1995). Implicit-explicit methods for reaction-diffusion problems in pattern formation.. J Math Biol.

[32] Turing A (1952). The chemical basis for morphogenesis.. Philos Trans R Soc Lond B.

[33] Koch AJ, Meinhardt H (1994). Biological pattern formation: from basic mechanisms to complex structures.. Rev Mod Phys.

