# Supplementary material for: A Dynamic Model for Stem Cell Homeostasis and Patterning in Arabidopsis Meristems
Source: PLoS One. 2010 Feb 12;5(2):e9189. doi: 10.1371/journal.pone.0009189 (PMC2820555; doi:10.1371/journal.pone.0009189)
Supplement: Text S1 — Reducing CLV3 expression. (0.03 MB RTF) [file pone.0009189.s006.rtf]

I. Reducing CLV3 expression
Conditional loss-of-function of CLV3 results in an expansion of the OC and the SCD. In addition, the OC is shifted towards the tip due to a loss of negative feedback by CLV3. When CLV3 expression levels are only mildly reduced, a graded response was observed: SCD and OC enlarge, but the location of the OC is not affected due to active CLV3 signalling. A typical time course of a simulation for a conditional reduction in CLV3 expression is shown in FigS1A. Interestingly, OC size remained unaffected for a wide range of endogenous CLV3 expression levels (Fig. S1B), indicating a partial uncoupling of OC and SCD sizes.
