# Supplementary material for: A Dynamic Model for Stem Cell Homeostasis and Patterning in Arabidopsis Meristems
Source: PLoS One. 2010 Feb 12;5(2):e9189. doi: 10.1371/journal.pone.0009189 (PMC2820555; doi:10.1371/journal.pone.0009189)
Supplement: Text S2 — Simulating loss-of-function mutants in CLV1. (0.86 MB RTF) [file pone.0009189.s007.rtf]

II. Simulating loss-of-function mutants in CLV1
The phenotype of clv1 loss-of-function mutants is comparable to a downregulation of CLV3 expression. Mathematically, a clv1 loss-of-function mutant can be incorporated by extending Eqs. 1 and 2 (see Materials and Methods section in the main article) with a factor  that modulates the response of WUS to CLV3 expression. The corresponding equations are given here:
		
		
By setting to values , different residual activity levels of CLV1 can be simulated, where  represents a loss-of-function, and  represents the wild type scenario. Typical simulated time courses for graded clv1 loss-of-function mutants are shown in Fig. S2.
