# Supplementary material for: A Dynamic Model for Stem Cell Homeostasis and Patterning in Arabidopsis Meristems
Source: PLoS One. 2010 Feb 12;5(2):e9189. doi: 10.1371/journal.pone.0009189 (PMC2820555; doi:10.1371/journal.pone.0009189)
Supplement: Text S3 — Gradual increase of exogenous CLV3 expression levels. (0.03 MB RTF) [file pone.0009189.s008.rtf]

III. Gradual increase of exogenous CLV3 expression levels
We reported simulations for meristems with strongly increased CLV3 expression levels, resulting in meristem arrest, and intermediate overexpression levels that caused an only transient loss of the SCD, which recovered with time. By studying a range of CLV3 overexpression levels, we uncovered that both domains are gradually reduced in size (Fig. S3A). However, the SCD (green line in Fig. S3B) responds more readily, indicating no strict interdependence of domain sizes.
