# Supplementary material for: A Dynamic Model for Stem Cell Homeostasis and Patterning in Arabidopsis Meristems
Source: PLoS One. 2010 Feb 12;5(2):e9189. doi: 10.1371/journal.pone.0009189 (PMC2820555; doi:10.1371/journal.pone.0009189)
Supplement: Text S4 — Altering WUS expression. (0.18 MB RTF) [file pone.0009189.s009.rtf]

IV. Altering WUS expression
We simulated the consequences of reducing WUS expression to different levels. Mathematically, a wus loss-of-function mutant can be incorporated by extending Eq. 1 and EQ. 2 (see Materials and Methods section in the main article) with a factor  that modulates the response of wus expression. The corresponding equations are given here:

	
By setting to values  different wus expression levels can be simulated, where  represents a loss-of-function, and  represents the wild type scenario. Minor reductions of WUS levels cause a corresponding small reduction in OC and SCD size (Fig. S4A). A medium reduction causes a transient loss of SCD and OC until facX reaches a level that allows to re-initiate a new meristem (Fig. S4B). In case of a WUS knockout, the meristem is arrested without recovery (Fig. S4C).
