# Supplementary material for: A Dynamic Model for Stem Cell Homeostasis and Patterning in Arabidopsis Meristems
Source: PLoS One. 2010 Feb 12;5(2):e9189. doi: 10.1371/journal.pone.0009189 (PMC2820555; doi:10.1371/journal.pone.0009189)
Supplement: Text S5 — Regeneration and de-novo generation of OC and SCD. (0.08 MB RTF) [file pone.0009189.s010.rtf]

V. Regeneration and de-novo generation of OC and SCD
New meristems can be formed during emryogenesis or flowering. Our model allows simulating the de novo generation of meristems by varying  regulation. Figure S5 shows the system response to different levels of the kinetic constant  and the resulting sizes of OC and SCD.  is varied over a range from 0.1 to 1, while  is used for wild type simulations.
