# Supplementary material for: A Dynamic Model for Stem Cell Homeostasis and Patterning in Arabidopsis Meristems
Source: PLoS One. 2010 Feb 12;5(2):e9189. doi: 10.1371/journal.pone.0009189 (PMC2820555; doi:10.1371/journal.pone.0009189)
Supplement: Text S6 — Sensitivity analysis for model parameters. (0.17 MB PDF) [file pone.0009189.s011.pdf]

VI. Sensitivity analysis for model parameters

We investigated the sensitivity of the proposed model with respect to perturbations of single parameters of the wild type parameter setting. The parameters have been perturbed using the following changes in absolute values: {-50%, -25%, -10%, 25%, 50%, 100%, 150%, 200%}.

The corresponding simulations document a considerable robustness with respect to these perturbations: apart from varying the five parameters ($\rho_a, \mu_a, \rho_{WUS_{sig}}, \mu_{WUS_{sig}}, K_{st}$), the simulated system response was qualitatively unchanged; only slight variations in domain size and degree of separation between OC and stem cell domain occurred.

ρ_a Variation

For a perturbation in ρ_a of -50%, OC and stem cell domain are no longer spatially separated but both are located in the tip. For variations of +150% or +200%, the functional domains are enlarged but separated, still during simulations small numerical instabilities occurred indicating that ρ_a is reaching level that becomes intractable by the current simulation setup. For the remaining perturbations the system remained qualitatively stable.

μ_a Variation

For a perturbations of +150% and +200% the formed domains are no longer spatially separated and located in the meristem tip. For the remaining perturbations the system remained qualitatively stable.

$\rho_{WUS_{sig}}$ Variation

With larger $\rho_{WUS_{sig}}$ levels domain sizes start to shrink and for perturbations of +100% to +200% simulations show small numerical instabilities. For the remaining perturbations the system remained qualitatively stable.

$\mu_{WUS_{sig}}$ Variation

For a perturbation of -50% domains are no longer spatially separated and both are located in the tip. This simulation shows small numerical instabilities as well. For perturbation levels of +150% and +200% simulations again no longer show spatial separation between functional domains, this time the simulations have been numerically stable. For the remaining perturbations the system remained qualitatively stable.

K_{st} Variation

Only for a perturbation of -50%, the system shows small numerical instability, but for all tested perturbation levels the system remains qualitatively stable.

Numerical Instabilities

As reported above, in 7 simulations of the sensitivity analysis instabilities occurred. In these cases, the simulations showed regular high-frequency oscillations with respect to time. There are two different possible explanations for the occurrence of these oscillations: (i) they could represent regular system behaviour which is rather improbable due to the fact that the oscillations are of high frequency and their exact patterns change slightly when varying the size of the time step used during integration. (ii) The used IMEX scheme for numerical integration employs a variant of the Crank-Nicolson integration scheme for the stiff diffusion terms. This modification was introduced to show better damping properties of the scheme with respect to false high-frequency oscillations [1]. Nevertheless, the observed oscillations could result from comparable false oscillations.

References

[1] Ruuth SJ (1995) Implicit-explicit methods for reaction-diffusion problems in pattern formation. J Math Biol 34: 148–176.
